# Toward the Relational Formulation of Biological Thermodynamics

**DOI:** 10.3390/e26010043

**Published:** 2023-12-31

**Authors:** Abir U. Igamberdiev

**Affiliations:** Department of Biology, Memorial University of Newfoundland, St John’s, NL A1C 5S7, Canada; igamberdiev@mun.ca

**Keywords:** attractor, autopoiesis, biological thermodynamics, kinetic perfection, relational biology, stable non-equilibrium

## Abstract

Classical thermodynamics employs the state of thermodynamic equilibrium, characterized by maximal disorder of the constituent particles, as the reference frame from which the Second Law is formulated and the definition of entropy is derived. Non-equilibrium thermodynamics analyzes the fluxes of matter and energy that are generated in the course of the general tendency to achieve equilibrium. The systems described by classical and non-equilibrium thermodynamics may be heuristically useful within certain limits, but epistemologically, they have fundamental problems in the application to autopoietic living systems. We discuss here the paradigm defined as a relational biological thermodynamics. The standard to which this refers relates to the biological function operating within the context of particular environment and not to the abstract state of thermodynamic equilibrium. This is defined as the stable non-equilibrium state, following Ervin Bauer. Similar to physics, where abandoning the absolute space-time resulted in the application of non-Euclidean geometry, relational biological thermodynamics leads to revealing the basic iterative structures that are formed as a consequence of the search for an optimal coordinate system by living organisms to maintain stable non-equilibrium. Through this search, the developing system achieves the condition of maximization of its power via synergistic effects.

## 1. The Incompleteness of Classical Thermodynamics for Describing Living Systems

Classical thermodynamics takes as a reference frame the state of thermodynamic equilibrium with the situation of maximal disorder or maximal disorganization of the constituent particles. From this state, the definition of entropy is derived, and the Second Law is formulated as describing the natural process of the tendency of all systems to reach the state of equilibrium. In classical thermodynamics, it was problematic to interpret the process of self-organization and complexification of natural systems. To develop the framework for understanding the process of order generation, non-equilibrium thermodynamics introduces the analysis of the fluxes of matter and energy that are produced in the course of the general tendency to achieve equilibrium. Within this tendency, the fluxes generate order from chaos and form organized structures that can potentially develop into living systems.

Ilya Prigogine [1,2] derived the condition of non-equilibrium from the Second Law, and the closed state of thermodynamic equilibrium remains the basic attractor in his theory. In this regard, non-equilibrium thermodynamics follows the paradigm of classical thermodynamics. The closed state of thermodynamic equilibrium is an abstraction that cannot be present in its pure expression in nature. To establish the state of thermodynamic equilibrium, it is necessary to measure it, which introduces its uncontrollable disturbance. We can ignore this disturbance to a certain extent, but we cannot eliminate it completely. Thus, the state of thermodynamic equilibrium is an abstraction of a similar nature as the Newtonian absolute space-time.

The concept of a closed equilibrium system may be heuristically useful, but epistemologically, it is incorrect because of its non-observability. An understanding of this leads to the limitations of the concept of entropy. It also places the process of measurement at the center of our comprehension of the physical universe. Measurement is profoundly more fundamental than the equilibrium state in the scientific conceptual background. The equilibrium state, which is the basis of classical and the non-equilibrium thermodynamics, should be measured to be defined as the equilibrium. However, this is impossible, because measurement itself is a profoundly non-equilibrium process, disturbing the state of equilibrium if it ever existed.

The notion of measurement should be taken not anthropocentrically, but rather as a control of the whole system over its components, and the interactions between biological systems themselves can be regarded as observations [3]. According to von Bertalanffy [4,5], living organisms are open thermodynamic systems exchanging matter and energy with their environment. This assumes that non-equilibrium thermodynamics is applicable for the analysis of these systems. In this framework, the driving force of the non-equilibrium process is a change in Gibbs energy, and for analysis of biological systems, it is not enough to consider only entropy but also necessary to analyze the changes in enthalpy and Gibbs energy. The analysis of biological systems as open non-equilibrium systems clarifies many aspects of their operation [6,7,8]. However, the autopoietic (self-maintaining) nature of living systems [9] assumes that, being open to the fluxes of matter and energy, they are closed to efficient causation, as was defined by Rosen [10]. This means that they possess their own internal constraints of functioning and development. This results in the fundamental limitations of non-equilibrium thermodynamics in the description of living processes.

## 2. Time, Energy, and Irreversibility

The approach to the unity of time, energy, and irreversibility in its basic features arises in relation to the concept of time that was developed by Aristotle in “Physica” [11]. It is defined in the following way: “The time marks the movement, since it is its number, and the movement the time. Time is a measure of motion and of being moved, and it measures the motion by determining a motion which will measure exactly the whole motion, as the cubit does the length by determining an amount which will measure out the whole” [11] (“Physica” 4:12). The physical time flow that corresponds to the rate of physical processes is “the fundamental motion which is uniform and is measured” according to Aristotle. The extra dimension of time corresponds to the agential time, i.e., to “the time by which we measure” [11]. The whole representation of the world appears in such a way that both time flows are complementarily reflected in the real world. This means that Aristotle interpreted time as a measurement.

The idea that time flow corresponds to quantum measurement was introduced in my earlier paper [12], where I considered the fundamental nature of quantum measurement as the basic process of the world structure. The fundamental irreversibility of quantum measurement as actualization corresponds to the irreversibility of time. It generates also the multiplicity of events in the observed world, because actualization appears as the choice from multiple alternatives and they can be realized sequentially, and thus, time acts as the engine separating contradictory realizations [13]. The opposition of these contradictory realizations holds energy for future development with new actualizations and generates new possibilities in the Universe. This means that energy is derived from the measurement process and is attributed to the measuring device. While the role of consciousness in the measurements performed by an observer is assumed, the unconscious biological systems perform internal measurements as endo-observers with their internal self-referential encoding [14,15,16].

The question arises about the nature of actualization outside the systems that are closed to efficient causation [10], i.e., the autopoietic living systems, acting as observers. This is the fundamental problem for any concept within the foundations of physics. The observers appear as living subjects only at the highest level of organization, while at the physical level, they emerge as the dynamic units of complex reality, i.e., the units of dynamic change. It has been suggested to define these units as ontolons to emphasize their profoundly ontological rather than epistemological nature [16,17]. In “Metaphysica”, Aristotle [11] attempted to formulate physics as the study of beings that arise on their own, with regard to their being, and thus introduced the relational principle to the field. In the frame of the inertia principle of Galileo, who claimed the equality of all inertial systems, it became the basis of the scientific exploration of reality. In the early 18th century, Leibniz [18] viewed the world as a multiverse of individual units (monads) that perform a kind of internal “computation”, corresponding to the physical idea of measurement. The spatial order appears as a consequence of the communicatory arrangement between monads. In the early 20th century, Whitehead [19] suggested to view the world as a set of occasions of experience (momentary monads) engaged in the temporal/causal succession.

The representation of physics on the basis of intercommunicating agents [20,21] or ontolons [16] refers to a pattern of the physical world as a universe of individual occasions or actions that form the relational space-time [22]. These occasions represent the cases of single quantum measurement events or transactions, whose efficient cause (in the Aristotelian sense) cannot be defined externally but has certain physical limitations. The efficient causes always precede the effects that shape regular events in our world. The irreversible transactions define the unidirectional flow of time in the real world. The spatiotemporal configuration of the world is determined by the coordinate system choice made by observers within the Universe [23]. This principle, describing the world’s relational arrangement as a set of systems with an internal causation, represents a generalized principle of relativity. A particular consequence of this principle is the fact that the absolute maximum value of the speed of signal is related not to the absolute time-space but to the agent (or, in the physical reality, to the individual occasion event). Another consequence is represented by the standpoint that the causation is possible only if there exists a materialized analogy of the infinitesimal acquiring the finite value in order to preserve the ability to measure everything that happens in the world. This “atomization” in the sense of ancient Greek philosophy frees the world from the immobility that is logically postulated by Parmenides and proved by Zeno’s aporias (paradoxes).

The physical space of occasions appears as an objective structure because it possesses universal characteristics such as the gravitational constant, as well as the constants that describe the additional compact dimensions, which include the elementary charge and the fine structure constant. To this “objective” world, a unified subset of the laws that are common to all observers is applied, which is expressed in the formal logic that has a substantially epistemic nature. However, there is a necessity to refer to the world consisting of multiple acting ontological units (ontolons) that operate and communicate as observers [16]. These units appear as conscious subjects only at the highest level of cognitive organization. At the level of the physical world, they represent “occasions” in the sense proposed by Whitehead.

Ontolons operating as agents produce the actions that actualize information and have the minimum action value (Planck’s constant); they can communicate by exchanging signals that have the maximum speed (the speed of light), and they generate the field that defines their interrelation (the gravitational field and other fields). Ontolons represent the individual beings with an internal determinism, which is framed in such fundamental properties as the absolute speed of signal (relative to an ontolon and not to the absolute space) and the minimum action for sending this signal (Planck’s constant) [16,17]. The observed complex structure of the physical world can be a consequence of the individual actions that are shaped by the limits of the establishment of three spatial dimensions of the gravitational field and of several additional compact dimensions corresponding to other fundamental forces [24]. The universal substantial time may be referred to the Primary Ontolon [16] or to Aristotle’s “principle on which heaven and nature depend” [11] (“Metaphysica”, 7.1072b13–14).

## 3. Time and Quantum Measurement

An attempt to put time into the basis of physics was carried out by Nikolai Kozyrev [25,26]. However, this scientific program was not developed into a complete theory and is sometimes used in pseudoscientific explorations. Apparently, Kozyrev suggested that the time flow in the Universe is its basic substantial process that may be related to its background progression of actualization, in which the cause and the effect are separated by the minimum time and space intervals. In this way, it corresponds to the most universal motion in the Aristotelian sense, which we measure using our internal time, which has the same nature, but it is relational and functions as the measure of the physical motion. In this sense, living systems contain two dimensions of time [27]. The appearance of measurable time occurs in the systems that perform quantum measurements in a regular way, with low dissipation of energy [13,14,28,29]. In living systems, time represents an independent measurable variable due to internal reproducible changes. The system becomes an internal autonomous clock that distinguishes the past (memory), the present (life), and the future (anticipation based on the reproducible model), so the modeling and logic become possible within such system [30].

The measurement itself remains self-contradictory until we consider it as occurring with finite velocity. Otherwise, it will contain contradictory statements at the same moment in time. The unfolding of the contradiction of movement via finite velocity of observation propagation was introduced by Gunji [31]. It may correspond to the background velocity of temporal unfoldment in Kozyrev’s concept, which he tried to relate to the fine structure constant [25]. In this case, the velocity of *c/137* would be the maximal flow of the actualization process in the course of measurement, which is slowed down in systems with low dissipation according to the uncertainty ratio of “energy-time”. Finite velocity in measurement provides decoherence that may be described as a continuous (prolonged in time) measurement. The rate of this continuous measurement will correspond to the flow of the substantial time in Kozyrev’s mechanics.

Quantum measurement belongs not to physics but to metaphysics in the sense that Gödel numbering belongs to metamathematics [17]. The concepts of quantum measurements are non-verifiable in the sense of physical theory. In fact, there is no physical verification for any version of the quantum theory of measurement. These versions can be evaluated starting from the point of their operational validity. Ernest Nagel [32] defined measurement as the correlation of numbers with entities that are not numbers. Kurt Gödel, when he introduced the code to prove the non-decidability theorem, assigned the numbers to the undefined statements, which corresponds to an expansion beyond the existing definitions [33]. In the same way, in quantum mechanical measurement, a non-formal process of mapping physical events onto symbols takes place.

Measurement in quantum mechanics corresponds to actualization, i.e., to the mapping to real numbers in terms of Rosen [10]. In another way, it corresponds to the performance of work in which the potential state is actualized into the actual state. This means that to perform a measurement, it is necessary to spend energy by using the measuring device. Jacobs [34] considers the energy cost of measurement as a fundamental question whose solution has profound implications for the foundations of physics. The cost of measurement is attributed to the measuring device. Only when a measuring device has access to a zero-temperature reservoir will measurement require no energy. We will show later that, although this is not achievable, it can approximate when the measuring devices have very long times of relaxation corresponding to the so-called quantum non-demolition measurements [35]. Generally, to obtain the information, measuring devices spend the energy that is equal to the energy amount that a heat engine would spend to obtain the equivalent work value of the information [34]. This means that irreversible information processing cannot be carried out without some inevitable thermodynamic work cost [36]. The minimum cost is defined by Landauer’s principle [37], according to which the minimum energy needed to erase one bit of information is proportional to the temperature at which the system is operating and is defined by the formula *E* ≥ *k_B_T* ln2, where *k_B_* is the Boltzmann constant.

Yi and Kim [38] demonstrated that quantum measurements perform non-equilibrium work on the measured system with limiting characteristics. If the measured system relaxes back to its initial equilibrium state, the work is completely dissipated in the form of heat into a reservoir. The corresponding entropy increase in the reservoir is not smaller than the von Neumann entropy change generated during the course of the measurements, proving Landauer’s principle. Mohammady and Miyadera [39] showed that the unattainability of pure states implies that a unitary interaction between the measured system and a measuring apparatus can never implement an ideal projective measurement. This unattainability follows from the Third Law of thermodynamics, according to which the zero-entropy state would correspond to the absolute zero temperature. While the Third Law rules out some of the properties for all observables, others may be attained at different approximations of the conditions determined by the Third Law.

The approach to a new paradigm of science that will be able to understand the emergent creativity is being developed by Stuart Kauffman [40,41]. According to the outline of this new paradigm presented in Kauffman [40], the actualization resulting in the construction of space-time is preceded by fully non-local entangled coherent quantum variables, followed by the onset of locality via decoherence. The discrete space-time appears as a set of relations among the discrete actualization events, while “now” is the shared moment of actualization of one among the entangled variables when the amplitudes of the remaining entangled variables change instantaneously. The quantum arrow of time arises from the discrete, successive, episodic, irreversible actualization events, and it is recorded in the very structure of the space-time that is constructed, while Actual Time is a succession of two or more actual events. This paradigm aims to explain and describe the emergent creativity, which cannot be described by mathematics based on set theory or by writing or solving differential equations for the diachronic evolution. However, we can apply the non-trivial operation of Gödel numbering to understand the formal basis of this fundamental process [42,43,44].

## 4. The Relational Approach in Biology and Thermodynamics

An approach that avoids a referral to thermodynamic equilibrium for describing thermodynamic characteristics of living beings was suggested by Ervin Bauer in 1920 [45] and then developed in more detail in 1935 [46]. This approach is fundamentally different from that developed in thermodynamics of irreversible processes, which was introduced by Ilya Prigogine [2] and based on the universality of the Second Law. The non-equilibrium thermodynamics of Prigogine, as well as the synergetics framework developed by Hermann Haken [47], resulted in significant progress in the understanding of self-organization and introduced a formal theoretical background for the description of dynamic complex systems. However, these scientific fields cannot in principle substantiate the specificity of life, because of the fundamental limitations that are imposed by their basic principles. These principles do not consider the internal efficient causes in the Aristotelian sense, which are intrinsic to the phenomenon of life [10].

An attempt to rework and reconsider thermodynamic principles characterizing life was outlined in a very general way by Robert Rosen [10] within the framework of relational biology that he developed. In particular, he mentioned that a closed system in the sense of classical thermodynamics cannot autonomously tend to an organized state, or, contrapositively, that a system autonomously tending to an organized state cannot be closed. A living system is open to the fluxes of matter and energy, but it is closed in another way, exhibiting the closure to efficient causation. In this state, attractors such as thermodynamic equilibrium, as well as the notion of thermodynamic entropy, lose their validity, and the teleonomic attractor that corresponds to the efficiency of the dynamic organization has to be employed. One such attractor suggested by Rosen is “the discrepancy between the actual behavior of the system and that of the standard or model which we have chosen to characterize order (or any numerical function of this discrepancy)” as a measure of disorder instead of entropy [48] (p. 252). Such a standard can be related to the system efficiency and thermodynamically defined as the stable non-equilibrium state.

Robert Rosen did not make sufficient progress in this direction. He only briefly outlined the basics of the non-classical thermodynamic concept for biology, which avoids a reference to the closed equilibrium system and refers to the standard characterizing order. The approach to theoretical biology, developed by Bauer a long time before Rosen, clarifies the nature of this standard. Bauer’s theory can be placed within the framework of relational biology, which is the study of biology from the standpoint of the “organization of relations”, as defined by its founder Nicholas Rashevsky [49]. Relational biology focuses on how function dictates structural organization, whence material aspects are entailed [50]. It identifies non-randomness with entailment, i.e., a sequence is non-random to the extent that it can be recursively generated [48]. The entailed autopoietic structure, whose first model was suggested by Tibor Gánti in 1952 [51], exists in the state of dynamic non-equilibrium and continuously maintains this state via the loops of internal regulation. It is closed to efficient causation in the Aristotelian sense [10] and constrains itself within a localized subregion of its phase space [40,41,52]. We will discuss below whether the internal teleonomic constraint can be defined as the Fourth Law of thermodynamics.

## 5. The Problem of the Fourth Law of Thermodynamics

Many researchers have realized the incompleteness of classical thermodynamics in the application to complex systems, in particular to living systems. Before the introduction of non-equilibrium thermodynamics, this incompleteness was recognized more sharply, while after the formulation of the ideas of synergetics and thermodynamics of irreversible processes, many scientists were deceived by the illusion of applicability of these new fields of science not only for describing living processes but also for the formulation of foundations of theoretical biology.

Alfred Lotka [53], in 1922, introduced the principle of maximum power for non-ergodic systems, i.e., for those systems that visit only a tiny subset of their possible states and hence exhibit history in a deep sense. Later, Odum and Pinkerton [54] considered this principle to be the Fourth Law of thermodynamics, or, more precisely, the fourth principle of energetics in open system thermodynamics. A few years before Lotka, Ervin Bauer [45] formulated the principle of a stable (sustainable) non-equilibrium state, which is applicable only to living systems and represents their main characteristics. According to Bauer [45,46], living systems, at the expense of their free energy, constantly perform work to avoid equilibrium under the existing external conditions. The stable non-equilibrium state determines the capacity of a living system for self-adaptation and the power for changing functions during its adaptation to its environment. To maintain its internal structure, a living system continuously supports and regenerates itself by consuming energy, and then the energy of the structural forces is used to perform the external work. This means that the laws of thermodynamics remain valid in living systems, but they are constrained by the specific state and structure of living matter; thus, there is no discontinuity between physics, chemistry, and biology. Bauer used the idea of maximum power to explain evolution by formulating the idea that progressive evolution proceeds toward higher external work that is performed by the system [46]. Boundary conditions constrain the release of energy into a few degrees of freedom in non-equilibrium processes, and this is followed by the propagation of the thermodynamic work that builds structures and controls processes [55].

The process of building biological structures incurs informational constraints that direct and govern the organization of the system and generally have no direct thermodynamic cost [56]. These constraints within living organisms override random processes to produce an organized spatiotemporal arrangement of metabolic processes. Although the elementary constituents of biological systems initially act upon it in a random manner, the outcome in a constrained system is predictable within an organism and across organisms. This assumes that the process of evolution may occur with little or no thermodynamic cost [56]. The energetic balance of the system aiming for its survival does not necessarily correspond to the maximum power performance. In certain cases, the restriction of energy use can result in a better survival efficiency; however, a common strategy under competitive conditions is the maximization of the use of available energies. In the later formulation, to avoid misinterpretations, Brown et al. [57] reformulated the maximum power principle as the principle of self-organization for maximum empowerment. In this formulation, the principle becomes very similar to Bauer’s principle of the stable non-equilibrium state. Thus, the stable non-equilibrium principle of Bauer determines the sustainability and metastability of living systems and applies to them as a more general principle than the original maximum power principle. The Fourth Law can be interpreted in a way that upon external energy input, living systems construct themselves into a localized subregion of their ever-expanding phase space [52]. According to Kauffman [52], the Fourth Law is reduced to the Second Law in the case of a pre-stated, fixed, and closed phase space that does not expand.

In the formulation of the Fourth Law by Lotka [53], Odum [58], and Kauffman [52], it is applicable only to a certain set of systems, such as to non-ergodic systems in Lotka’s formulation and to living systems and the whole biosphere in Kauffman’s interpretation. The principle of the stable non-equilibrium state of Bauer [46], which can also be referred to as the Fourth Law, characterizes only living systems and determines their uniqueness. Since all these authors do not claim that living systems possess their own physical laws, their concepts rather refer to the main constraint of living systems than to an additional physical law. The difference between laws and constraints was definitively formulated by Howard Pattee [59]. In this sense, the Fourth Law is beyond physics and beyond thermodynamics, and is rather a constraint that does not violate physical principles but constrains them in living systems.

The problem of the applicability of the Second Law to living systems seems to be non-trivial and mainly based on misunderstandings [28,60]. The complexity of living systems cannot be simply derived from the external non-equilibrium. Living systems actively search for the external non-equilibrium milieu that is suitable for their existence according to their internal constraints. The active search of living systems for complexity goes beyond the thermodynamic principles. The principles of thermodynamics are the most arbitrary among all physical principles: they work in the actualized non-living (non-generic in the terms of Rosen [10]) world. That is why the concept of a stable non-equilibrium state, generated by the system itself [46], seems most valuable to overcome the trivialities of the thermodynamic approach to describe living systems.

The Newtonian paradigm requires a pre-stated, fixed phase space. A similar fixed space is the equilibrium system of classical and non-equilibrium thermodynamics. According to Kauffman [52], living cells and organisms are Kantian Wholes that achieve constraint closure and carry out thermodynamic work to construct themselves. They also undergo the process of evolution, which creates an ever-expanding phase space: at a constant energy input, the biosphere can construct itself into an ever more localized subregion of its expanding phase space [52]. To understand the distinction of living systems from the inanimate world, the principles have been introduced that are sometimes referred to as the Fourth Law. However, we should rather define these principles as constraints that are inherent to living systems, because these principles do not expand the set of physical laws but constrain their operation.

The laws of thermodynamics outline different levels of causality in the world. The basic causality referred to as the conservation of energy corresponds to the First Law. The dynamic causality in the fixed phase space, which exhibits itself in the increase of entropy, is described as the Second Law. The Third Law defines the highly ordered attractor of the zero entropy. The approximation of its achievement can occur through the teleonomic causality, which, however, represents a constraint of all living systems and is sometimes referred to as the Fourth Law, although it is rather an organizing principle that would be incorrect to consider the law of thermodynamics.

## 6. Internal Causation and Subzero Temperatures

The self-controlled operation of biological systems toward the most efficient utilization of energy becomes possible due to their effective ascendency by the hierarchically dominant subsystem over all internal subsystems, efficiently shielded from energy flows. This represents an internal epistemic cut [61], complementing the recognition of external low stimuli and being reached in highly ordered coherent states within the system [14,29,60,62]. The function of nucleic acids that store the information and thus maintain the field of meanings, representing the potential for development and adaptation of a particular biological system, makes possible the persistence and reproduction of biological organization as an invariant entity. The subsystem controlling the whole system to provide an efficient and precise control is shielded from the heat motion and has a physical reference. Descartes anticipated such a physical subsystem by associating it with epiphysis, and to the same framework belong the concepts of coherence in microtubules [63], of the quantum regulator [64], of the internal quantum state [13,29], and of the “eidos-navigator” [65].

In living systems, the function cycle (“Funktionkreis”) [66] is implemented internally via the system of reflective loops generating the network of biological codes. This becomes possible in the frames of the fundamental structure, which was originally called “chemoton” by Gánti [51] and which is also defined as the (M,R) system by Rosen [10] and as the autopoietic structure by Varela et al. [67]. Although the representations of this structure in different concepts have certain variances, they all have in common the self-causation and self-maintenance [68]. In this metabolically closed structure, which may represent the basic feature of life, its elements fulfil the role of a “double duty”, serving both as energetic and as informational components that are equilibrated through the balance of energy and information fluxes. The meaning of information that is held by the system corresponds to the most optimal and efficient way of utilizing energy in order to achieve the system’s optimal performance.

The network of biological macromolecules maintains the internal ordered potential state as shielded from thermal fluctuations, which can be referred to as the “internal quantum state” (IQS) [29]. Its most coherent part corresponds to perceptive actions and conscious activity. Vladimir Lefebvre [65] defined it as the “eidos-navigator”, which governs the rest of the body by perceiving the whole system, sending commands to it and ensuring that the organism operates with maximum power and efficiency. These coherent internal states are experienced by the system as qualia. Understanding this would lead us to approach the “hard problem of consciousness” [69], which actually refers not only to consciousness but rather to any information-bearing systems, e.g., to any self-perception of the observer. The original idea of Dubrovsky [70], who formulated it explicitly in the 1960s, that the informational processes in the brain are inseparable from the qualia allows us to assume the existence of the protophenomenal properties in any natural observer such as a living cell or organism or even in any transactional measurement event in the physical world.

To understand the operation of biological systems through the governance by the eidos-navigator, we can introduce the concept of the temperature of the highly ordered internal coherent quantum state. This temperature reflects the kinetic energy of the quantum states making up a coherent field, and it can be determined through the dissipation of energy from this internal quantum state. This dissipation of energy occurs via the emission of photons during the conformational relaxation of biomacromolecules, e.g., in the enzymatic catalysis or the transfer of genetic information. This follows from Heisenberg’s uncertainty ratio of “energy-time”, as shown in my earlier paper [14]. In fact, the operation of biomacromolecules as quantum-measuring devices [71,72] corresponds to them performing the quantum non-demolition measurements [35], in which the long relaxation times of the measuring device provide the conditions of low energy dissipation and maintenance of prolonged coherent states.

An attempt to determine the temperature of coherent states during the contraction of the actomyosin complex was performed by Matsuno [73,74]. He showed that actin-activated myosin ATPase activity functions as a heat sink operating effectively at an extremely low temperature and that the extraction of heat energy from the actin filament condenses the atomic degrees of freedom constituting the filament into a macroscopic quantum state carrying a non-vanishing linear momentum. The sliding movement of an actin filament on myosin molecules while hydrolyzing ATP molecules is a consequence of the quantum mechanical coherence due to an extremely slow release of the energy stored in an ATP molecule. Matsuno demonstrated that actomyosin ATPase activity is associated with emission of a quanta of energy that is 2.2 × 10^−19^ erg or 1.6 mK in temperature. Thus, the effective temperature of an actomyosin complex in the presence of ATP molecules comes to decrease down to 1.6 × 10^−3^ K [73,74,75]. It has a much lower temperature than the cosmic background radiation (2.725 K). The stable non-equilibrium acts as a refrigerator that supports this low-temperature state. In the cytoskeletal assemblies, the conformational relaxation expands to the distances of the cellular and intercellular macroscales. It is possible that the most ordered processes associated with perception and consciousness correspond to effective temperatures of a lower range, maybe of several orders of magnitude (the nanokelvin range or less). The idea of a low dissipation and subzero temperature of a coherent state follows from my paper [14].

In fact, the idea that most ordered living processes are shielded from the heat motion was introduced in the famous book of Erwin Schrödinger “What is Life”. Schrödinger [76] stated that “…the aperiodic crystal forming the hereditary substance, <is> largely withdrawn from the disorder of heat motion”. This means that living order is realized close to an absolute zero temperature and that Nernst’s Third Law of thermodynamics is more important for understanding the phenomenon of life than the Second Law, which is valid in the case of a pre-stated, fixed, and closed phase space that does not expand [52]. The idea of shielding of ordered living processes from the heat motion was considered by several researchers, among them Nikolai Kobozev [77] for enzymatic catalysis as well as for mental phenomena and Iosif Rapoport [78] for the transfer of genetic information. The organizational condition for this shield is the stable non-equilibrium state principle of Ervin Bauer [45,46]. For understanding life, the Third Law of thermodynamics appears as the most essential principle, being more important than the Second Law, which cannot explain specific principles of the living state. Colin McClare [79] and Lev Blumenfeld [80] introduced the concept describing the non-equilibrium mechanism of conformational relaxation of biomacromolecules and of recuperation of energy in this process. According to these views, the physical mechanism of the elementary act of enzyme catalysis represents a slow conformational relaxation occurring upon the fast substrate binding at the active site, during which a transformation of the substrate molecule into a molecule of product takes place. Blumenfeld [81] experimentally detected the out-of-equilibrium states in several iron-containing proteins; new studies developed and confirmed these ideas [82].

The Third Law states that the points of the state space of zero temperature are adiabatically inaccessible from the state space of a simple system [83]. This implies the unattainability of absolute zero by a finite number of operations, i.e., in a finite time; however, using sufficiently long times of quantum measurement, it is possible to approach the close-to-zero state in which all points are adiabatically equivalent with a high approximation. Thus, the Third Law of thermodynamics is essential for understanding the efficient function of living systems. In fact, the principle of approximation to zero temperature via the maintenance of quantum coherence within a heat engine is that physical principle, which Erwin Schrödinger [76] introduced in his famous book.

## 7. Relational Biology and Spatiotemporal Rescaling

Biological systems continuously transform their energy flow structure in a way so that the useful energy transformation becomes maximized via the constrained release of energy that delays the production of entropy [55]. This occurs at different scales which include the adaptation to a changing environment, the process of individual development, and the large-scale evolutionary process. Living systems can be analyzed via such criteria as the productivity, efficiency, and profitability of various mechanisms for capturing and utilizing energy to build biomass and perform work [84,85]. The continuous transformation of a biological structure occurs via the spatiotemporal rescaling [29]. This is based on the alterations of the morphogenetic coordinate system, as described by D’Arcy Thompson [86], and can be based on parametrization of the morphogenetic field (the normalization principle), as suggested by Gurwitsch [87]. Such parametrization can occur in morphogenesis via the alterations of differentiation trees [88,89]. Mikhailovsky and Levich [27] called this omnicausality to emphasize its difference from the bottom-up causality characteristic of classical physics. The omnicausal laws of biological systems appear as the constraints that provide a frame for the operation of physical laws without violating them.

If we abandon the absolute space, as in, the foundations of the theory of relativity, the Euclidean space is transformed into the non-Euclidean spatiotemporal continuum. Similarly, in biological systems, the relational space cannot be Euclidean. The relational thermodynamic approach, which abandons the reference frame of the equilibrium system and refers to the perfection of the final state, aims to describe the non-Euclidean shaping of living beings. As a consequence, the morphology is formed via the non-classical causality when establishing the relationally stable non-equilibrium state, as described by Petukhov [90], who presented examples of iterations based on biochemical and autocatalytic cycles that lead to the formation of particular morphogenetic structures. This establishment occurs via the application of internal iterative algorithms that operate in living systems and develop not in the planar Euclidean space but through the adjustment of curvilinear trajectories [91].

Biological systems, in their adaptation, development, and evolution, as well as in the perception of external signals, adjust their coordinate scales in accordance with the principle of their optimal operation, which at the cellular level is based on the rearrangement of the cytoskeleton [92]. The developmental process unfolds as a search for the goal-directed (teleonomic) coordination that is fully established at the adult stage. The adult stage is also a matter of natural selection [93,94]. Liberman [92,95] outlined the principle of optimality of a coordinate system to expand the principle of relativity to the relational processes occurring in living systems. The principle of relativity in physics does not take into account the influence of measurement and computation on the process. The realization of any biological function corresponds to the establishment of an optimal coordinate system for this function. An example suggested by Liberman [92] is the rotating body, which requires solving the problems of maintaining balance during rotation. The adjustment of coordinated scales may occur in multiple ways that include metabolic tuning, channels and transporters functioning, second messengers’ synthesis, ubiquitin and proteasome activation, and fundamentally enhanced gene expression, plus possible cell migration and potential cytoskeleton arrangement, meaning that the case described by Liberman [92] emphasizes just one of those instances.

The principle of maximum useful energy flow transformation, by substantiating the kinetic perfection of biological systems as their evolutionary attractor, determines the durability of their non-equilibrium state and thus their competitiveness in the process of adaptation to environment. The hypercyclic structure that underlies these properties is based on the active search towards fitting the slow non-equilibrium processes and the fast equilibration reactions into the metabolic structure [96]. In fact, the equilibria of coenzyme nucleotides and substrates that are established in cells generate simple rules that provide optimal conditions for the non-equilibrium chemodynamic fluxes of major metabolic processes. The equilibrium “buffering” enzymatic reactions serve as control gates for the non-equilibrium fluxes through the “engine” enzymes providing the directed diffusion of substrates to their active sites and establishing the balance of the fluxes of load and consumption of metabolic components. The search for the best “buffering” configurations for “engine” enzymes represents an optimized strategy in the developmental trajectories. The evolutionary optimization is achieved under the coordinated operation of the buffering and the engine enzymes that determine the establishment of stable and organizationally invariant non-equilibrium states that organize the fluxes of energy spatially and temporally by controlling the rates of major metabolic fluxes that follow thermodynamically and kinetically defined computational principles [96].

Relational biological thermodynamics aims to abandon the abstract state of thermodynamic equilibrium and to exploit the reference frame that is relative to a biological function operating within the context of a particular environment. The stable non-equilibrium state is realized via the mutual resonant self-synchronization of many physiological processes in a living body [97]. This process represents a temporal cohesion that underlies semiosis and generates biological order [98,99] via the retrocausal action [100]. The explanation based on temporal cohesion and retrocausality represents an alternative to the explanation via negentropy. The latter, which arises as a response to Schrödinger [76], has a very limited value for living organisms, as it uses the standard of the equilibrium system that is fully applicable only to non-generic physical bodies [10]. Using the non-equilibrium thermodynamics framework, it was shown that some microorganisms exhibit enthalpy-retarded microbial growth, and that entropy-neutral, entropy-driven, and entropy-retarded instances of growth exist in nature [101].

The basic structures of living bodies are formed as a consequence of the active search for an optimal coordinate system by biological systems teleonomically realizing their internal stable non-equilibrium state. The relational principles in biology appear as an extension of the physical principles of relativity. The internal establishment of an optimal coordinate system is a consequence of the maintenance of the essential thermodynamic non-equilibrium, which represents the basic process, relative to which all living activities are arranged [102,103].

## 8. The Evolutionary Process as a Codepoiesis

The fixed number of elements characterizes the equilibrium system that is used as a reference standard in classical and non-equilibrium thermodynamics. For the evolving biological systems and the biosphere as a whole, this standard cannot be defined, because the number of elements of the phase space is not fixed (increasing). Kauffman and Roli [41] emphasize the impossibility of defining or deducing the evolving phase space or of writing or solving differential equations for the diachronic evolution of ever-new adaptations. The new elements appear in this evolution as previously undefined code statements that expand the system and open new possibilities in its adaptation and development. The origin and evolution of life corresponds to physical phase transitions that are associated with the emergence of a new type of grand canonical ensemble and a corresponding new level of description [104].

The phase space of life expanded through the evolution of genes and metabolism [105]. The ability to enhance the rate of core reactions creates an energetic basis for the selection of subsequent layers of biological complexity [106]. This ability was realized through the linking of the transmembrane electrochemical potential difference of hydrogen ion concentration to the synthesis of the macroergic compound (ATP), which represents a gated proton semiconductor that carries protons and allows them to interact specifically with well-defined substrate molecules [107]. These features of living organization, which include the biosynthetic pathways leading to simple metabolites, the structures of organic and metal ion cofactors, homochirality, and template-directed replication of nucleic acids, which arose in prebiotic systems and were retained in the course of evolution, and in which living systems developed into more sophisticated complex structures [105].

The self-maintenance of a system via its internal regulation supports its stable non-equilibrium state and denotes its autopoietic organization [9,67,108]. A transformation beyond the limits of an autopoietic system results in its complexification. Marcello Barbieri [109,110] defined the basis of this transformation as a “codepoiesis”. As biological systems realize internal computation [91,92], the codes underlying biological organization possess a capacity to grow up through the attainment of indefinite statements within them by meaning, which corresponds to the process of proving the incompleteness theorem. This idea relates to my earlier paper [42], and it was developed in consequent publications [43,44,111].

The whole process of generating novel coding statements that substantiate organizational complexification leads to the expansion of the system that often incorporates the elements that originally were external to it. As a result, a newly generated complex structure arise. During this complexifying process, new meanings are assigned to the previously unproven and thus previously “meaningless” statements. The set of meanings in the biological semiotic system has its limits, but it also has a property of overcoming these limits, and this corresponds to evolution. According to Gödel’s incompleteness theorem, any sufficiently powerful consistent logical system has true statements, which cannot be proven within its framework. In relation to biological systems, this assumes that the foundation of these statements exists outside the formal language of the system, being imposed through the creative process of evolutionary transformation. In the transformative process, the elements of a formal system acquire meanings via the procedure defined as Gödel numbering. In this procedure, new semiotic relations emerge that can attain interpretations in the formalized language. Codepoiesis appears to be an intrinsic property of biological systems through the mapping of a living system as a whole into the finite set of its molecular structures [43].

Codepoiesis occurs in multiple ways, which all involve unique and novel significations by using new codes or rearranging the old ones through the irreversible reduction in a fundamental uncertainty in the self-referential living process. New traits in evolution originate as “exaptations”, appearing as the features that are available for useful cooptation by descendants [112,113]. The exaptation configurations become empowered by meaning in the timeline of evolution. At the time of their origin, they do not possess a definitive functional role, which they gain in the course of temporal development. The whole evolutionary process leads to an increase in the kinetic perfection of biosystems and shows itself in the rates of metabolic fluxes, in the spatial movement of organisms, and in their mechanical structure including hydro- and aerodynamic characteristics [114]. The kinetic perfection as a part of the power maximization of the system determines the choice of a unique efficient trajectory in the multidimensional space of biological evolution.

The semiotic pattern of a living system opens the possibilities for potentially infinite evolutionary unfolding of codepoiesis. The operational features that include negative feedbacks, feedforward links, equifinality, and flexibility toward external influences can be viewed as final causes (in the Aristotelian sense) in this evolutionary development. They represent the far-from-equilibrium attractors of a dynamical system, achieved with the participation of particular mechanisms, preventing possible deviations in development from various external perturbations [115,116,117]. For the emergence of novel genetic changes, the mobile genetic pool of all organisms serves as a source of horizontal gene transfer [118], and this genetic pool represents a part of the biosemiosphere that is common for all living beings. The biosemiosphere is not restricted only to genetic elements comprising all symbolic elements that include digital-, index- and analog-coded structures. All these structures attain symbolic functions in the processes of intercommunication and evolutionary complexification.

## 9. Conclusions

The paradigm discussed here and defined as relational biological thermodynamics refers to the standard that relates to a biological function operating within the context of a particular environment and not to the abstract state of thermodynamic equilibrium. This standard corresponds to the stable non-equilibrium state in accordance with the concept of Ervin Bauer [46]. Relational biological thermodynamics reveals the fundamental iterative structures emerging via the non-equilibrium chemodynamic biological processes that are formed through the teleonomic search by living organisms for an optimal scale to maintain stable non-equilibrium. The analysis of biological systems within the framework of non-equilibrium thermodynamics successfully clarifies many aspects of their operation, but their autopoietic nature remains largely unexplained on these terms. While living systems are open to the fluxes of matter and energy, they possess their own internal constraints in their operation, development, and the adaptation to the environment, which imposes fundamental limitations on the non-equilibrium thermodynamics in the description of living processes.

The relational concept of biological thermodynamics is focused on the internal biological causality governing the self-maintenance and development of living systems. The standard to which it refers represents the actual biological function that is realized in the context of a particular environment. Living systems, being never in equilibrium, continuously perform work at the expense of their free energy to avoid the equilibrium under existing external conditions. They possess the ability to actively transform the external fluxes of energy into the work against the equilibrium to support the basic properties of biological systems such as adaptability, expediency, regulation, and integrity. Living systems achieve the condition of maximization of their power via synergistic effects in their development and evolution by the maintenance of their autopoietic structure and its expansion in the process of codepoiesis.

## Data Availability

No data were generated for this study.
